# Psychometric validation of the internet related experiences questionnaire and mobile related experiences questionnaire among Ecuadorian teenagers

**DOI:** 10.3389/fpsyg.2024.1390174

**Published:** 2024-06-05

**Authors:** Livia I. Andrade, Marlon Santiago Viñán-Ludeña, Carmen Sanchez

**Affiliations:** ^1^Department of Psychology, Universidad Técnica Particular de Loja, Loja, Ecuador; ^2^Escuela de Ingeniería, Universidad Católica del Norte, Coquimbo, Chile; ^3^Departamento de Ciencias de la Computación e Inteligencia Artificial, ETSI Informática y de Telecomunicación, CITIC-UGR, University of Granada, Granada, Spain

**Keywords:** validation, reliability, internet addiction, smartphone addiction, Ecuador, IREQ, MREQ

## Abstract

**Introduction:**

Excessive internet and mobile cell phone use has been increasing in recent years especially in teenagers who are a vulnerable population. However, there is a lack of psychometric evaluation of instruments that allow to identify behavior regarding problematic use of the internet and cell phones in the Latin America, particularly in Ecuador. The main aim of this study is to examine the psychometric properties of two instruments: the Internet-related experiences questionnaire (IREQ) and Mobile-related experiences questionnaire (MREQ) in high school students (*n* = 4, 174, *M*_*age*_ = 15.63; 51.19% male and 48.37% female).

**Methods:**

The validation process was performed using one and two factors for both questionnaires according to previous literature. After checking the models proposed to date, the best fit model was the one-factor model for (IREQ) and one-factor model for (MREQ).

**Results and discussion:**

The invariance was performed using two samples according to gender (male, female) and has been confirmed with an acceptable internal consistency for both questionnaires. For IREQ (**All**, ω = 0.80; **Male**, ω = 0.77; **Female**, ω = 0.82) and for MREQ (**All**, ω = 0.83; **Male**, ω = 0.82; **Female**, ω = 0.84). In addition, we performed the correlation analysis between IREQ, MREQ, and socio-demographic variables and finally, both instruments demonstrated strong psychometric qualities within the local population.

## 1 Introduction

Addiction, according to the literature, is a persistent and problematic behavior related to the use or abuse of substances or behaviors, characterized by the inability to control them. The word “addiction” today is used to refer to a chronic condition in which there is a powerful and unhealthy motivation to engage in a particular behavior. This can be due to many factors, such as physiological, psychological, environmental, and social (West and Brown, 2013; La Sociedad Española de Patología Dual et al., 2017; Rios, 2019).

The World Health Organization (WHO) and the American Psychiatric Association (APA), (2013), both include non-substance related disorders in their latest version of the DSM-5. These disorders include behavioral addictions, which activate the reward system in a similar way to drugs. The main symptoms of a behavioral addiction are: (1) an intense desire, craving, or unstoppable need to carry out the pleasurable activity; (2) a progressive loss of control over the activity, leading to uncontrollable behavior; (3) neglect of previous habitual activities, such as family, academic, work, or leisure time; (4) negative consequences that are usually noticed by people close to the addict, who may become defensive and deny the problem; and (5) a progressive focus on the addiction, leading to neglect or abandonment of previous interests and relationships. Additionally, irritability and discomfort may occur when attempting to abstain from the addictive behavior or when unable to stop after a short period of time.

Today, the most pertinent behavioral addictions involve excessive internet usage and smartphone dependence, characterized by compulsive and repetitive behaviors leading to severe consequences for those affected. These behaviors stem from engaging in pleasurable or satisfying activities, often resulting in challenging-to-control habits (Pedrero Pérez et al., 2017; Rodríguez et al., 2023; Tejada Garitano et al., 2023).

Thus, the Internet and cell phones are the most used technologies in people's daily lives. Worldwide, 66.2% of the population uses the Internet, and 69.4% of the population uses cell phones (Kemp, 2024). They are increasingly used by adolescents, a population considered vulnerable because it is a very difficult period of life due to psychological, physical, and social changes (Buitrago Ramírez et al., 2020; de Freitas Moreira et al., 2021; McGivern and Reilly, 2021; Regalado Chamorro et al., 2022).

Therefore, it can be said that both the internet and mobile devices serve primarily as facilitators of interpersonal communication among adolescents, yet they offer a variety of leisure services, information searches, and communication via chat applications. Notably, the cellphone stands out as having the most significant influence on individuals' social relationships, particularly among adolescents (Dou et al., 2020; Fang et al., 2020; Yang et al., 2023). Regarding gender differences, usage patterns reveal that women tend to utilize cellphones more frequently than men do, whereas men typically engage more with the internet (Kawyannejad et al., 2019). Literature suggests that parental control acts as a safeguard against excessive or unhealthy internet usage within families (Salinas et al., 2021; Rodrigues et al., 2022). Studies have indicated that urban environments exhibit higher levels of internet and cellphone availability, potentially leading to increased instances of problematic device usage in those regions. Lastly, recent research has demonstrated a positive correlation between resilience and academic achievement, alongside an inverse association between internet addiction and scholar success (Sánchez-Yarmas, 2020).

The excessive use of the internet and cell phones has generated consequences in all areas, as visualized in greater detail during the COVID-19 confinement. These consequences include cognitive and mental health impacts (Anderson et al., 2016; Chu et al., 2021), such as irritability, stress, anxiety, and depression in adolescents (García-Oliva et al., 2017; Pedrero Pérez et al., 2017; Díaz-Vicario et al., 2019; Del Prete and Pantoja, 2020; Díaz et al., 2020; Ozturk and Ayaz-Alkaya, 2021; Servidio et al., 2021; Kumar et al., 2022; Xie et al., 2023). Additionally, symptoms of prefrontal malfunction, especially in memory failures and higher cognitive functions, have been observed. On the other hand, surfing the web causes hyper-exposure to people in general, creating a false social status that is often used as a facade to conceal reality, leading to feelings of loneliness and guilt internally. In conclusion, the high frequency of cell phone use causes damage to health. In this sense, there are studies that indicate a relationship between addiction to cell phone use and blood pressure and heart rate (Amiri et al., 2022). Other research concludes that there is a relationship between addiction to cell phone use and poor sleep quality (Tettamanti et al., 2020; Mohamed and Moustafa, 2021; Sohn et al., 2021).

Other pathologies resulting from improper cell phone usage include nomophobia, phubbing, and FOMO (Al-Saggaf and O'Donnell, 2019; Durak, 2019; Díaz Miranda and Extremera Pacheco, 2020; Ivanova et al., 2020; Aydin and KuÅÝ, 2023; Jiang et al., 2023; Rahmillah et al., 2023). As such, adolescents exhibiting high levels of cell phone addiction tend to experience anxiety over losing their devices, fear missing out on events, and disregard others (family, friends) while focusing on their mobile phones.

Within the Ecuadorian populace, only a limited number of investigations have examined this issue, as by the year 2023, Ecuador's overall population amounted to ~18.10 million individuals, among whom 81.3 percent utilized the internet and 92.3% relied on mobile connectivity (Kemp, 2023), this underscores the necessity for a trustworthy and standardized tool capable of assessing behaviors related to problematic internet and cellphone usage across both clinical and non-clinical settings.

Both questionnaires were selected for their validity and precision. The MREQ enables the assessment of intra- and interpersonal conflicts associated with cell phone use and incorporates diagnostic criteria from the DSM. In terms of sensitivity and specificity analysis, a Likert scale is utilized to enhance instrument sensitivity and specificity, aiding in distinguishing individuals with addiction issues from those without. Additionally, while many tools for evaluating behavioral changes linked to Internet and mobile phone use are in English, the IREQ and MREQ are designed in Spanish, eliminating the need for translation into the Ecuadorian context.

Previously, the Internet-Related Experiences Questionnaire (IREQ) and the Mobile-Related Experiences Questionnaire (MREQ) have been validated in Spain, Italy, and Argentina. These findings are presented in Table 1. Nevertheless, the psychometric properties in Ecuador remain unknown, which makes both evaluation and research difficult. Therefore, the aim of the present study is to analyze the psychometric properties and factorial structure of the (IREQ) and (MREQ), tests developed by Beranuy Fargues et al. (2009), in a considerable sample of Ecuadorian adolescents.

**Table 1:** Summary of studies about IREQ and MREQ.

**References**	**Country**	**Age**	**Factors**	**Format**	α	**Sample**
Ruscio and Stover (2023)	Argentine	18–76	MREQ (C; CEU)	Likert (4)	0.69, 0.66	541
Servidio et al. (2021)	Italy	14–20	IREQ (IaC, IC)	Likert (4)	0.66, 0.65	438
Casas et al. (2013)	Spain	12–18	IREQ (IC, IaC)	Likert (4)	0.72, 0.64	525
Fargues et al. (2009)	Spain	12–25	IREQ (IC, IaC), MREQ (C, CEU)	Likert (4)	IREQ (0.77), MREQ (0.80)	1,879

C, conflicts; CEU, communication and emotional use; IaC, intrapersonal conflicts; IC, interpersonal conflicts.

## 2 Materials and methods

### 2.1 Participants and procedure

This research is a scale adaptation study that involves several steps to ensure that both IREQ and MREQ are appropriate for use in the Ecuadorian context. To summarize, we first determined that conceptual equivalence is relevant, as mentioned earlier in this manuscript. Additionally, translation and content review were not necessary because we considered the work of Beranuy Fargues et al. (2009) and both questionnaires are in the Spanish language. Subsequently, to validate comprehension of the items, a pilot study was carried out using a modest sample size. Later, we conducted a larger study collecting a big sample (the sample was obtained by non-probabilistic convenience sampling) to perform a comprehensive psychometric evaluation, including exploratory factor analysis, confirmatory factor analysis, and measures of reliability and validity. Finally, we conducted the analysis taking into account the results and conclusions of this research.

This study was conducted following ethical guidelines for research in psychology. First, a research protocol was designed by Andrade and Ontaneda (2015), which helped psychology students of the “Universidad Técnica Particular de Loja”, apply the questionnaire to students in the 10th grade of general basic education, and in the first- and second-year secondary school, from public and private institutions, in 76 cities in Ecuador such as Quito, Cuenca, Loja, Guayaquil, etc. Subsequently, the participants were informed that their participation is voluntary and confidential. The directors of the educational centers, the parents and/or legal representatives and the teenagers signed the informed consent and assent form prior to the execution of the objectives of research. Lastly, data were collected in the classrooms of educational institutions during 2016,^1^, using non-parametric incidental sampling techniques; thus, data collection depended on the availability of the respondents. The study was approved by the vice-director of research of the “Universidad Técnica Particular de Loja”.

### 2.2 Instruments

We applied a socio-demographic test as an *ad hoc* survey designed for data collection related to the main aim of this study. It is composed by five short-answer questions which collect data about: age, gender, family type, school dropout and neighborhood.

Internet-related experiences questionnaire IREQ (Fargues et al., 2009), is a 10-item self-administered instrument that assesses internet abuse. The authors of this questionnaire found two factors: intrapersonal conflicts (items 4, 5, 6, 7, 9, 10) and interpersonal conflicts (items 1, 2, 3, 8). The response score varies between 1 (not at all) and 4 (very much); the higher the score, the higher the probability of having a problematic use of the internet.

Mobile-related experiences questionnaire MREQ (Fargues et al., 2009). This is a 10-item self-administered instrument that assesses problematic cell phone use. The questionnaire is organized into two factors (conflicts and communicational-emotional use). The response score varies between 1 (not at all) and 4 (very much). The higher the score, the higher the probability of having cell phone use.

### 2.3 Ethics

This research was done following the ethical guidelines for research in psychology through a protocol developed by Andrade and Ontaneda (2015). In addition, this study followed the ethical principles included in the Declaration of Helsinki (de los Angeles Mazzanti Di Ruggier, 2011) in its updates, and in the current codes and guidelines such as: consent, personal data protection, confidentiality, non-discrimination, gratuity, and the possibility to drop out the study. On the other hand, the students were informed that their participation is voluntary and confidential. Furthermore, the parents and/or legal representatives signed the informed consent, and the students signed the informed assent, prior to the execution of the research objectives. Finally, the respondents were voluntary, confidential, and anonymous.

### 2.4 Data analysis

Screening data is necessary before the validation process, removing missing data, duplicate cases, and other anomalies. Afterwards, the Mahalanobis Distance was performed to identify multivariate outliers within dataset with a threshold value of *p* < 0.001 (Hu and Bentler, 1999; Andrade et al., 2022). Finally, Mardia's multivariate skewness and kurtosis were calculated to examine if the IREQ and MREQ items had multivariate normality (Mardia, 1970), see Tables 3, 4.^2^ In addition, the descriptive statistics is shown in Table 2.

**Table 2:** Socio-demographic characteristics of the sample.

	**n**	**%**
**Gender**		
Male	2,137	51.19%
Female	2,019	48.37%
Other	18	0.43 %
**Family type**		
Single parent	929	22.26%
Both parents (nuclear)	2,392	57.31%
Extended family	447	10.71%
Others	406	9.73%
**School dropout**		
Yes	487	11.67%
No	3,687	88.33%
**Neighborhood**		
Urban	3,237	77.55%
Suburbs	937	22.45%

The Kaiser-Meyer-Olkin (KMO) is used to measure sampling adequacy which shows a measure of factorability (Kaiser and Rice, 1974); according to the guidelines the cutoff selected for determining the factorability of the dataset is *KMO* ≤ 0.60. Subsequently, to identify the structure of IREQ and MREQ, the exploratory factor analysis was performed to determine the number of factors.

In the scale adaptation study, exploratory factor analysis (EFA) is not mandatory, but we included it to examine the scale's structure. We evaluated the original two-factor model, but it yielded an unacceptable fit. Conversely, the one-factor model produced an acceptable fit.

In order to validate the IREQ and MREQ among Ecuadorian students, the Confirmatory Factor Analysis was performed using R package (Lavaan) (Rosseel, 2012) using maximum likelihood estimation to test structural model. Multisampling-modeling was conducted to investigate the IREQ and MREQ test measurement invariance regarding to participants gender (male and female). The goodness fit of the model was considered acceptable according to five indicators: (1) Chi-square *p*−*value*>0.5; (2) Comparative Fit Index > 0.90; (3) Tuker-Lewis fit Index (TLI) > 0.90 (Hu and Bentler, 1999); (4) Root Mean Square Error of Approximation (*RMSEA*) < 0.06 with its 90% confidence interval and (5) Standardized Root Mean Squared Residual (*SRMSR*) < 0.08 (Browne and Cudeck, 1992).

To examine the model invariance across the male and female samples, we conducted further tests on metric (factor loading) invariance scalar (measurement intercept) invariances in the model (Andrade et al., 2022). The overall invariance model was fitted with an equal factor structure, across two samples, and factor loadings and measurement intercepts to be freely estimated. Then, two restrictive invariance models were fitted to evaluate the invariance across samples. Lastly, comparisons were performed with the ratio test and the change in CFI and, to evaluate the invariance, the following criteria were considered: (i) the χ^2^ difference between the most restrictive models (e.g., metric invariance, scalar invariance) are not significantly different from the less restrictive, baseline model (configural invariance) with α < 0.05 (optional); (ii) the change in *CFI*≥0.01 (mandatory), and (iii) the change in *TLI*≥0.02 (optional). This process was conducted for both questionnaires.

Concurrent validity was tested using correlational analysis, computing Crombach' alpha to ensure the internal consistency. In addition, we also compute the McDonald's omega due to criticism of Crombach' alpha (Malkewitz et al., 2023).

## 3 Results

The sample is composed by 4,174 participants, who were 14- to 18-years old (M = 15.63, SD = 1.02, female = 48.37%). All participants reported that they have cell phone and had used internet before. All the descriptive statistics are described in Table 2.

The sample was divided into two groups for both questionnaires. The first group consisted of 2,087 participants and was used for exploratory factor analysis (EFA). The second group, consisting of 2,087 participants, was used for confirmatory factor analysis (CFA).

### 3.1 IREQ

The validity of the IREQ was checked by exploratory and confirmatory factor analysis. The KMO test and Barlett's test produced satisfactory results. The residual matrix did not exceed 10% and the KMO coefficient of 0.89 shows that the matrix was of optimum suitability. To identify the underlying structure of the IREQ we utilized factor analysis using maximum likelihood and the proportion of explained variance is 0.286.

The results obtained by exploratory factor analysis show only one factor in contrast to the original solution proposed by Fargues et al. (2009), composed by two-factor model (intra-personal and inter-personal conflicts); thus, the item distribution and variance percentage differed from the original IREQ as showed in Table 3.

**Table 3:** Exploratory factor analysis, factor solution (IREQ).

**Items**	**M**	**SD**	**Skewne**	**Kurtosis**	**Loadings**	***h* ^2^**	***r***	**α**
**Factor 1: inter and intrapersonal conflicts**								
Item 7	1.87	0.85	0.72	0.43	0.433	0.19	0.29	0.78
Item 8	1.77	0.77	0.67	0.59	0.633	0.40	0.27	0.77
Item 9	1.82	0.87	0.69	0.10	0.434	0.19	0.29	0.79
Item 1	1.78	0.96	0.92	0.05	0.520	0.27	0.28	0.78
Item 2	1.85	0.95	0.66	−0.10	0.503	0.25	0.29	0.78
Item 3	1.94	1.05	0.71	−0.53	0.553	0.31	0.28	0.78
Item 4	1.75	0.93	0.95	0.29	0.549	0.30	0.28	0.77
Item 10	1.39	0.78	1.77	3.15	0.598	0.36	0.27	0.77
Item 5	2.58	1.06	−0.11	−0.88	0.582	0.34	0.28	0.77
Item 6	1.79	0.93	0.91	0.18	0.511	0.26	0.28	0.78

**Figure 1:**
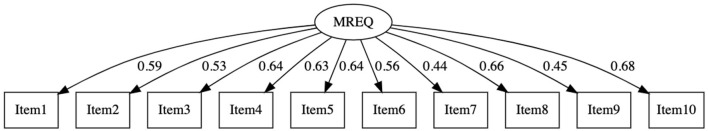
Confirmatory factor analysis of the IREQ. All factor loadings were significant (*p* < 0.01).

Confirmatory factor analysis corroborated the model obtained in the exploratory factor analysis. We tested a model with only one factor, this model showed an acceptable fit: χ^2^(35) = 180.946, *p* < 0.001; *CFI* = 0.990; *TLI* = 0.987; *RMSEA* = 0.045, 90% CI [0.038,0.051]; *SRMR* = 0.035 and the factor loadings were satisfactorily high (0.44–0.68). As presented in Figure 1, all items significantly loaded on the single factor.

In addition, the two-factor model was also tested. This model showed the following fit: χ^2^(34) = 298.915, *p* < 0.001; *CFI* = 0.991; *TLI* = 0.988; *RMSEA* = 0.043, 90% CI (0.039, 0.048); *SRMR* = 0.032 and the factor loadings were between (0.44–0.65). Also, to compare the two models we calculated the Akaike information criterion (AIC). The AIC value for bi-factor model was 103,535.6 while the AIC value for one-factor model was 51,850.79, thus, we selected the one factor model.

### 3.2 MREQ

First, we test the validity of the MREQ. The KMO and Barlett's tests also produced satisfactory results. The residual matrix did not exceed 10% and the KMO coefficient of 0.89 shows that the matrix was of optimum suitability. In the same manner, to identify the underlying structure of the MREQ we utilized factor analysis using maximum likelihood and the proportion of explained variance is 0.31.

After performing the exploratory factor analysis the results show only one factor in contrast to the original solution developed by Fargues et al. (2009), composed by two-factor model (conflicts and communication-emotional use); thus, the item distribution and variance percentage differed from the original MREQ as showed in Table 4.

**Table 4:** Exploratory factor analysis, factor solution (MREQ).

**Items**	**M**	**SD**	**Skewne**	**Kurtosis**	**Loadings**	***h* ^2^**	***r***	**α**
**Factor 1: conflicts and communication-emotional use**								
Item 1	1.33	0.66	1.97	4.11	0.48	0.23	0.32	0.80
Item 2	1.56	0.75	1.21	1.39	0.55	0.30	0.31	0.79
Item 6	1.42	0.74	1.73	2.79	0.56	0.32	0.31	0.79
Item 3	1.43	0.76	1.59	2.45	0.53	0.28	0.31	0.80
Item 4	1.40	0.71	1.76	3.29	0.69	0.47	0.29	0.78
Item 9	1.73	0.95	1.05	0.30	0.59	0.35	0.30	0.79
Item 10	1.62	0.86	1.16	1.01	0.62	0.38	0.30	0.79
Item 5	1.19	0.57	1.66	1.98	0.49	0.24	0.32	0.80
Item 7	2.52	1.06	−0.002	−0.99	0.50	0.25	0.32	0.80
Item 8	1.88	0.94	0.78	−0.12	0.55	0.30	0.31	0.79

**Figure 2:**
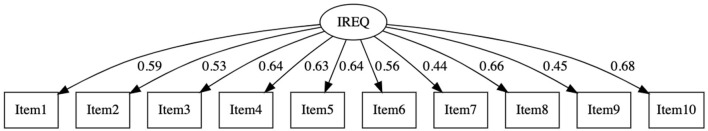
Confirmatory factor analysis of the MREQ. All factor loadings were significant (*p* < 0.01).

Confirmatory factor analysis was performed to test the model obtained in the exploratory factor analysis. The model with one factor model showed an acceptable fit: χ^2^(35) = 180.946, *p* < 0.001; *CFI* = 0.990; *TLI* = 0.987; *RMSEA* = 0.060, 90% CI (0.054, 0.066); *SRMR* = 0.035 and the factor loadings were satisfactorily high (0.44–0.68). As presented in Figure 2, all items significantly loaded on the single factor.

In addition, the two-factor model was also tested. This model showed the following fit: χ^2^(34) = 298.915, *p* < 0.001; *CFI* = 0.991; *TLI* = 0.988; *RMSEA* = 0.043, 90% CI (0.039, 0.048); *SRMR* = 0.032 and the factor loadings were between (0.58–0.65). Furthermore, to compare the two models we calculated the Akaike information criterion (AIC). The AIC value for one-factor model was 51,850.79 while the AIC value for bi-factor model was 90,369.5, thus, we selected the one factor model.

### 3.3 Factorial invariance across female and male samples

Tables 5, 6 shows the factor loadings of each item across whole group as well as across samples for IREQ and MREQ tests.

**Table 5:** Factor loadings of the IREQ test, Chronbach's alpha α and McDonald's omega ω.

**IREQ**	**Male (ω = 0.77, 0.75)**	**Female (ω = 0.82, α = 0.79)**	**All (ω = 0.80, α = 0.77)**
**Factor 1-Interpersonal conflicts**			
‘Con qué frecuencia haces nuevas amistades con personas conectadas a Internet? (How often do you make new friends in the internet?)(Item 7)	0.42	0.40	0.40
‘Con qué frecuencia abandonas las cosas que ests haciendo para estar más tiempo conectado a la red? (How often do you give up things you are doing in order to be connected for longer?) (Item 8)	0.67	0.64	0.66
‘Piensas que tu rendimiento académico o laboral se ha visto afectado negativamente por el uso de la red? (Do you think your academic and professional performance have been negatively affected due to internet use?) (Item 9)	0.46	0.40	0.41
Cuando tienes problemas, ‘conectarte a Internet te ayuda a evadirte de ellos? (When you are in trouble, does getting online help you to escape from them?) (Item 1)	0.58	0.52	0.55
‘Con qué frecuencia anticipas tu próxima conexión a la red? (How often do you anticipate your next connection?) (Item 2)	0.53	0.51	0.52
‘Piensas que la vida sin Internet es aburrida, vacía y triste? (Do you think life without internet is boring, empty and sad?) (Item 3)	0.61	0.61	0.61
‘Te enfadas o te irritas cuando alguien te molesta mientras estás conectado? (If someone disturbs you while you are connected, do you get angry or irritated?) (Item 4)	0.65	0.56	0.61
‘Cuando no estás conectado a Internet, te sientes agitado o preocupado? (When you are not connected to the Internet do you feel nervous or worried) (Item 10)	0.73	0.67	0.70
‘Cuando navegas por Internet, te pasa el tiempo sin darte cuenta? (When you are navigating through the internet, do you feel time flies?) (Item 5)	0.66	0.58	0.62
‘Te resulta más fácil o cómodo relacionarte con la gente a través de Internet que en persona? (Do you find relating to people through internet easier or more convenient than face to face?) (Item 6)	0.55	0.52	0.53

**Table 6:** Factor loadings of the MREQ test, Chronbach's alpha α and McDonald's omega ω.

**MREQ**	**Male (**ω = 0.82, α = 0.80**)**	**Female (**ω = 0.84, α = 0.81**)**	**All (**ω = 0.83, α = 0.81**)**
**Factor 1-conflicts (disputes)**			
‘Has tenido el riesgo de perder una relación importante, un trabajo o una oportunidad académica por el uso del móvil? Have you ever been at risk of losing an important relationship, a job, or an academic opportunity because of cell phone use? (Item 1)	0.58	0.54	0.56
‘Piensas que tu rendimiento académico o laboral se ha visto afectado negativamente por el uso del móvil? Do you think your academic or work performance has been negatively affected by cell phone use? (Item 2)	0.59	0.60	0.60
‘Hasta qué punto te sientes inquieto cuando no recibes mensajes o llamadas? To what extent do you feel restless when you do not receive messages or calls? (Item 6)	0.59	0.62	0.60
‘Sufres alteraciones de sueño debido a aspectos relacionados con el móvil? Do you suffer from sleep disturbances due to mobile-related issues? (Item 3)	0.61	0.59	0.60
‘Sientes la necesidad de invertir cada vez más tiempo en el móvil para sentirte satisfecho? Do you feel the need to spend more time on your cell phone to feel satisfied? (Item 4)	0.75	0.79	0.77
‘Piensas que la vida sin el móvil es aburrida, vacía y triste? Do you think life without a cell phone is boring, empty, and sad? (Item 9)	0.66	0.69	0.67
‘Te enfadas o te irritas cuando alguien te molesta mientras utilizas el móvil? Do you get angry or irritated when someone bothers you while you are using your cell phone? (Item 10)	0.68	0.70	0.69
‘Dejas de salir con tus amigos por pasar más tiempo utilizando el móvil? Do you stop hanging out with your friends because you spend more time using your cell phone? (Item 5)	0.60	0.57	0.58
Cuando te aburres, ‘utilizas el móvil como una forma de distracción? When you're bored, do you use your cell phone as a form of distraction? (Item 7)	0.56	0.62	0.59
‘Con qué frecuencia dices cosas por el móvil que no dirías en persona? How often do you say things on your mobile that you wouldn't say in person? (Item 8)	0.60	0.58	0.59

In order to have evidence that both tests have the same meaning across different groups, in Tables 7, 8 we performed the test of invariance for both IREQ and MREQ.

**Table 7:** Tests of invariance for IREQ questionnaire.

	**Model fit**						**Model comparison**				
**IREQ**	χ^2^	**df**	**CFI**	**TLI**	**RMSEA [90% CI]**	**SRMR**	Δχ^2^	Δ*df*	Δ*CFI*	Δ*TLI*	Δ*RSMEA*
Configural	568.859	108	0.985	0.987	0.045	0.035					
Weak metric	385.958	79	0.99	0.988	0.043	0.036	182.901	29	0.005	0.001	0.002
Strong scalar	568.859	108	0.985	0.987	0.045	0.035	182.901	29	0.005	0.001	0.002
Factor inv.	757.762	109	0.978	0.982	0.054	0.038	188.903	1	0.007	0.005	0.009

CFI, comparative fit index; TLI, Tucker–Lewis index; RMSEA, root mean square error of approximation; SRMR, standardized root mean square residual; CI, confidence interval.

**Table 8:** Tests of invariance for MREQ questionnaire.

	**Model fit**						**Model comparison**				
**MREQ**	χ^2^	**df**	**CFI**	**TLI**	**RMSEA [90% CI]**	**SRMR**	Δχ^2^	Δ*df*	Δ*CFI*	Δ*TLI*	Δ*RSMEA*
Configural	563.735	108	0.986	0.988	0.045	0.045					
Weak metric	528.132	79	0.986	0.984	0.052	0.046	35.603	29	0.00	0.004	0.007
Strong scalar	563.735	108	0.986	0.988	0.045	0.045	35.603	29	0.00	0.004	0.007
Factor inv.	606.028	109	0.985	0.987	0.047	0.045	42.293	1	0.001	0.001	0.002

CFI, comparative fit index; TLI, Tucker–Lewis index; RMSEA, root mean square error of approximation; SRMR, standardized root mean square residual; CI, confidence interval.

Analyzing IREQ results, the configural invariance shows a good model fit: χ^2^(108) = 568.859, ρ < 0.001; *RMESA* = 0.045; *CFI* = 0.985; *TLI* = 0.987; *SRMR* = 0.035. The metric invariance also had a good model fit: χ^2^(79) = 385.958, ρ < 0.001; *RMESA* = 0.043; *CFI* = 0.99; *TLI* = 0.988; *SRMR* = 0.036. The scaled Δχ^2^(29) = 182.901, ρ < 0.001, Δ*CFI* = 0.005; δ*RSMEA* = 0.002. There was a little change in CFI (< 0.01) indicating that the metric invariance model was maintained across samples; meanwhile, the scalar invariance model has does not change in comparison with the configural model. Thus, our results have shown that both factor loadings and measurement intercepts were equal across male and female samples.

MREQ results show that the configural invariance indicates a good model fit: χ^2^(108) = 563.735, ρ < 0.001; *RMSEA* = 0.045; *CFI* = 0.986; *TLI* = 0.988; *SRMR* = 0.045. The metric invariance also had a good model fit: χ^2^(79) = 528.132, ρ < 0.001; *RMSEA* = 0.052; *CFI* = 0.986; *TLI* = 0.984; *SRMR* = 0.046. The scaled Δχ^2^(29) = 35.603, ρ < 0.001; Δ*CFI* = 0.00; Δ*RSMEA* = 0.007. There was no change in CFI indicating that the metric invariance model was maintained across samples; meanwhile, the scalar invariance model has does not change in comparison with the configural model. Thus, our results also have shown that both factor loadings and measurement intercepts were equal across male and female samples.

Models were evaluated to have significant change in goodness of fit compared between configural, metric, scalar and factor invariance when two of three criteria are met: the χ^2^ difference was significant (ρ < 005) for both tests, the change in CFI between configural-metric and metric-scalar was equal to 0.005 while between scalar and factor invariance was equal to 0.007 for IREQ and no change was shown for MREQ between configural-metric and metric-scalar while the change between scalar and factor invariance was equal to 0.001; the change in TLI was equal to 0.001 between configural-metric and metric-scalar while the change between scalar and factor invariance was equal to 0.005 for IREQ. For MREQ the change in TLI between configural-metric and metric-scalar was equal to 0.004 while the change between scalar and factor invariance was equal to 0.001; finally, the RSMEA was equal to 0.002 (configural-metric and metric-scalar) and 0.009 (scalar and factor invariance) for IREQ and 0.007 (configural-metric and metric-scalar) and 0.002 (scalar and factor invariance) for MREQ, which is in concordance with Chen (2007) that it should be < 0.015.

### 3.4 Reliability and concurrent validity

According to Cronbach's alpha and McDonald's Omega shown in Tables 5, 6 the reliability for both IREQ and MREQ were high as overall as well as for male and female samples.

The correlations between and IREQ and socio-demographic variables [i.e., gender, family type, schooling status (dropout) and neighborhood] were explored to verify concurrent validity are shown in Table 9. There were no correlation between IREQ and the variables: Gender (ρ = −0.005, *p* < 0.001), family type (ρ = −0.0002), school dropout (ρ = 0.003, *p* < 0.001) and and neighborhood (ρ = 0.000)

**Table 9:** Concurrent and convergent validity of the IREQ test.

	**1**	**2**	**3**	**4**	**5**
1. IREQ	–	−0.005^**^	−0.0002	0.003^**^	0.000
2. Gender	–	–	−0.000	0.005^**^	0.000
3. Family Type	–	–	–	0.000^*^	0.000
4. School dropout	–	–	–	–	0.000
5. Neighborhood	–	–	–	–	–

^*^ρ < 0.005); ^**^ρ < 0.001).

In the same manner the correlations between MREQ and socio-demographic variables [i.e., gender, family type, schooling status (dropout) and neighborhood] were explored to verify concurrent validity. There were no correlation between MREQ and the variables: Gender (ρ = −0.05), family type (ρ = 0.000), school dropout (ρ = 0.001, *p* < 0.05) and neighborhood (ρ = 0.000).

## 4 Discussion and conclusions

The main objective of this study was to validate the Internet-related experiences questionnaire (IREQ) and the Mobile-related experiences questionnaire (MREQ) using data from Ecuadorian students. Overall, the results suggest that both IREQ and MREQ can serve as reliable and valid assessment tools in the Ecuadorian-speaking population.

First of all, the single-factor structure of the IREQ and MREQ was cross-validated in a CFA, which was consistent with the findings of previous studies (Fargues et al., 2009; Casas et al., 2013; Servidio et al., 2021). These unidimensional solutions were consistently reported across different gender groups (Andrade et al., 2022). These findings support the conclusion that both questionnaires exhibit a fairly robust factor structure.

Our factor structure suggests that both questionnaires do not align with the proposed models from the original study in Spain. This discrepancy may be attributed to cultural differences (Huang et al., 2007) or cognitive aspects (Davis, 2001). Nevertheless, the questionnaires still enable the assessment of the inappropriate use of the internet and mobile devices.

There are two important theoretical reasons why we used the one-factor model. Firstly, the principle of parsimony suggests that the simplest model that adequately fits the data should be preferred. Furthermore, there is limited prior theory or empirical evidence supporting a multi-factor structure for these specific questionnaires in the Ecuadorian context. Thus, a one-factor model may be a reasonable starting point for analysis in this context.

The reliability analysis demonstrated excellent internal consistency for both questionnaires (IREQ: α = 0.77; MREQ α = 0.81) reaching the values reported in the original study (IREQ: α = 0.77; MREQ α = 0.80) (Fargues et al., 2009). Furthermore, we conducted an exploratory factor analysis with one and two factors. The results indicated that the best-fitting models were one-factor models for both questionnaires. Thus, the obtained results demonstrate that the questionnaires are reliable, possess validity, and contribute to the knowledge about IREQ and MREQ for future research in Ecuador and Latin America.

In relation to IREQ, the items (Item1, Item2, Item3, Item4, Item6, and Item10) are associated with focusing, avoiding, denial, and other cognitive distortions. All of these items are considered crucial for diagnosing Internet addiction (Beard and Wolf, 2001; Tsai and Lin, 2001; Young, 2007). However, upon evaluating the results obtained in this study, we found that respondents, on average, answered these items as “Sometimes”. The other items (Item5, Item7, Item8, and Item9), which are linked to social-related conflicts, are also considered criteria for addiction (Wang, 2001; Huang et al., 2007; Young, 2007), with respondents, on average, answering as “Sometimes”. Regarding MREQ, the addiction criteria differ, as it pertains to the excessive use of various applications (both on- and off-line) installed on mobile phones, leading to impairment in physical, psychological, and social functions (Zhang et al., 2023). Upon evaluating our results, the average value assigned to each of the items corresponds t “Sometimes”.

In addition, our results for internet and cell phone behavioral addiction show a low prevalence in accordance with previous studies (Fargues et al., 2009). Thus, for IREQ, 36.73% of the respondents shown an addictive internet prevalence and for MREQ, 20.51% of the respondents shown an addictive cell phone prevalence. Whereas, men and women do not differ in their behavioral addictive use of the Internet and the cell phone in contrast by another studies, women tend to cell phone abuse utilizing as a channel of communication and expression of emotions (Kubey et al., 2006; Jenaro et al., 2007). This work will be able to clinically evaluate and effectively diagnose the internet and cell phone addiction on clinical and non-clinical samples.

This study was limited to a certain extent by factor originating in its very design, such the time elapsed between data collection and the publication of results and the social desirability bias inherent to such questionnaires as reported in the original and Spanish versions (Fargues et al., 2009). In addition, This study not evaluate how well the questionnaire aligns with other established measures of the same construct, which could have provided additional evidence of the questionnaire's validity and reliability. In the future we will compare other questionnaires that measure the same construct to assess convergent validity.

Future research might include other disorders and psycho-social variables such as cognitive distortion and problem gambling studied by Labrador Encinas and Villadangos González (2010) and look at their possible relationship with the internet and cell phone abuse. In addition, we believe that more research should be done on how social media can influence with the internet and cell phone behavioral addiction specially in young people with the aim to prevent those possible risks with the implementation of action models.

## Data availability statement

The raw data supporting the conclusions of this article will be made available by the authors, without undue reservation.

## Ethics statement

The studies involving humans were approved by Ethics Committee for Research in Human Beings (Comité de Ética de Investigación en Seres Humanos, Hospital de Especialidades “Eugenio Espejo”, June 3, 2015) and the vice-rectorate for research of the UTPL Ecuador (PROY-PSC-1302). The studies were conducted in accordance with the local legislation and institutional requirements. Written informed consent for participation in this study was provided by the participants' legal guardians/next of kin. Written informed consent was obtained from the individual(s) for the publication of any potentially identifiable images or data included in this article.

## Author contributions

LA: Visualization, Resources, Project administration, Investigation, Funding acquisition, Writing – review & editing, Writing – original draft, Validation, Methodology, Conceptualization. MV-L: Software, Formal analysis, Data curation, Writing – review & editing, Writing – original draft, Validation, Methodology, Conceptualization. CS: Writing – original draft, Writing – review & editing, Project administration, Methodology, Funding acquisition, Conceptualization.

## Footnotes

^1^The data belong to a longitudinal project; due to administrative problems there was a delay in data collection and, due to problems generated by COVID-19, we are presenting the results few years later.

^2^**Skewness** values between −2 and +2 are generally considered acceptable for normality and **Kurtosis** values between −7 and +7 are generally considered acceptable for normality.
